# Latent class modeling to compare testing platforms for detection of antibodies against the *Chlamydia trachomatis* antigen Pgp3

**DOI:** 10.1038/s41598-018-22708-9

**Published:** 2018-03-09

**Authors:** Ryan E. Wiegand, Gretchen Cooley, Brook Goodhew, Natalie Banniettis, Stephan Kohlhoff, Sarah Gwyn, Diana L. Martin

**Affiliations:** 1 Division of Parasitic Diseases and Malaria, Centers for Disease Control and Prevention, Atlanta GA, USA; 2 State University of New York Downstate Medical Center, Brooklyn, NY USA

## Abstract

Latent class modeling can be used to combine the results of multiple tests to compare the sensitivity and specificity of those tests in the absence of a gold standard. Seroepidemiology for chlamydia infection may be useful for determining the cumulative risk of infection within a population. Initial studies using the *Chlamydia trachomatis* immunodominant antigen Pgp3 have shown utility for seroepidemiology of sexually transmitted chlamydia and the eye disease trachoma. We present our latent class modeling results for comparison of antibody data obtained from three different Pgp3-based platforms – multiplex bead array, ELISA, and lateral flow assay. Sensitivity and specificity estimates from the best fitting latent class models were similar to estimates derived from those previously obtained using a nucleic acid amplification test as a gold standard for sensitivity and non-endemic pediatric specimens for specificity, although the estimates from latent class models had wider confidence intervals. The modeling process and evaluation highlighted the importance of including as many antibody tests as possible when fitting a latent class model to ensure that as many patterns as possible are available for evaluation. Future studies designed to evaluate antibody test performance in the absence of a gold standard should utilize as many tests as possible.

## Introduction

For many diseases, a “gold standard” test, i.e., an evaluation which clearly defines disease status, is not available or is cost-prohibitive. However, disease status based on a single test may provide biased estimates of an individual’s disease status or a test’s performance^1^. Latent class analysis is commonly used to combine results from multiple, imperfect tests, provided that those tests follow an assumption of conditional independence^2,3^. A latent class model can be used to classify participants by disease status, to estimate disease prevalence, or to estimate test accuracy. The use of latent class analysis for combining information from multiple diagnostic tests has become more common, particularly in the area of infectious diseases^3,4^.

At present, there is no gold standard for serologic testing for the bacterium *Chlamydia trachomatis* (CT), the etiologic agent of sexually transmitted chlamydia and the eye disease trachoma. Serologic outcomes may have utility in estimating the cumulative risk of CT infection for surveillance^5–8^. Serology for CT is focused on detection of antibodies to the CT protein plasmid gene product 3 (Pgp3). Pgp3 is an immunodominant antigen in urogenital infection^9^ and is under evaluation in serosurveillance studies for trachoma^6,10,11^. Three platforms exist for detecting antibodies to Pgp3: multiplex bead array (MBA); enzyme-linked immunosorbent assay (ELISA), and lateral flow assay (LFA)^12^.

The performance of these platforms has been reported using ocular swab nucleic acid amplification test (NAAT)-positivity as the gold standard for infection and non-endemic controls to determine specificity^13^. In these analyses, sensitivity was estimated at 93.2% for ELISA and MBA, and specificity ranged from 96.1% to 99.4% for the various assays. This approach has some limitations, particularly because ocular infection may be detectable before an individual produces an antibody response. We therefore used latent class analysis to compare these platforms. Originating in the social sciences^14^, latent class analysis (or latent class modeling) analyzes multivariate categorical data (also called indicators) which are related to an underlying latent construct. For diagnostic test studies without a gold standard, that construct is disease status. Without a gold standard, we are unable to observe a person’s true disease status. Instead, there are multiple tests (i.e. the indicators) which should relate to the underlying latent variable of a person’s disease status, though none directly measure the disease status. Hence, latent class analysis tries to leverage multiple tests to arrive at a best guess of each person’s probability of membership in each latent class.

Our goals are two-fold: (1) to report the performance of each of the antibody tests utilized here using a latent class model; and (2) to highlight this application of latent class model, especially to demonstrate how certain choices impact model estimates. Despite the use of latent class modeling for evaluating diagnostic tests, few applied examples exist detailing the modeling process. Thus, we hope to help others fit more robust latent class models and to understand some of the aspects of this modeling approach.

## Results

Three models fit the data noticeably better than the other models: Models 17, 22, and 23 (Table 1, bold font). Those three all possessed non-significant p-values for each of the fit tests. A fourth model (Model 18) also had p-values greater than 0.05 for each test (G^2^_(6)_ = 9.03, p = 0.09; χ^2^_(6)_ = 10.81, p = 0.17), but given the potential that these goodness-of-fit tests are underpowered and these p-values are much closer to 0.05 than for other tests, we have chosen not to include it with the other three.

**Table 1:** Sensitivity and specificity of tests measuring antibodies to Pgp3 based on input parameters. Pos = positive; ind = indeterminate; neg = negative. LFA = lateral flow assay; ELISA = enzyme-linked immunosorbent assay; MBA = multiplex bead array. ROC = receiver operator characteristics; MM = mixture model. DF = degrees of freedom. AIC = Akaike Information Criterion. BIC = Bayesian Information Criterion. Bolded rows are referenced in the text.

Model #	Latent Classes	Diagnosis Categories	LFA Blood test	LFA Serum test	ELISA coating	Cutoff	N	DF	AIC	BIC	G^2^ test p-value	χ^2^ test p-value	Proportion positive	MBA Sensitivity	MBA Specificity	ELISA Sensitivity	ELISA Specificity	LFA Sensitivity	LFA Specificity
1	2	Pos/neg	Yes	Yes	Fresh	ROC	283	4	743.6	783.7	0.11	< 0.01	0.36 (0.28–0.44)	0.95 (0.87–1.00)	0.98 (0.96–1.00)	0.87 (0.77–0.96)	0.98 (0.94–1.00)	0.87 (0.77–0.97)	0.96 (0.91–1.00)
2	2	Pos/neg	Yes	Yes	Dried	ROC	283	4	736.6	776.7	< 0.01	< 0.01	0.35 (0.27–0.43)	0.96 (0.90–1.00)	0.98 (0.95–1.00)	0.81 (0.69–0.92)	0.99 (0.97–1.00)	0.88 (0.79–0.98)	0.95 (0.90–1.00)
3	2	Pos/neg	Yes	Yes	Fresh	MM	283	4	742.2	782.2	0.08	0.01	0.37 (0.29–0.45)	0.92 (0.84–1.00)	0.99 (0.96–1.00)	0.99 (0.96–1.00)	0.91 (0.85–0.98)	0.85 (0.75–0.95)	0.97 (0.93–1.00)
4	2	Pos/neg	Yes	Yes	Dried	MM	283	4	692.1	732.2	0.14	0.03	0.37 (0.29–0.45)	0.93 (0.85–1.00)	0.98 (0.96–1.00)	0.99 (0.96–1.00)	0.96 (0.92–1.00)	0.86 (0.76–0.96)	0.97 (0.93–1.00)
5	2	Pos/neg	No	Yes	Fresh	ROC	569	0	1247.1	1277.5			0.42	0.97	0.98	0.89	0.98	0.99	0.97
6	2	Pos/neg	No	Yes	Dried	ROC	569	0	1266.8	1297.2			0.41	0.98	0.98	0.86	0.98	0.99	0.96
7	2	Pos/neg	No	Yes	Fresh	MM	569	0	1210.2	1240.6			0.43	0.95	0.99	1.00	0.92	0.98	0.98
8	2	Pos/neg	No	Yes	Dried	MM	569	0	1144.8	1175.2			0.43	0.95	0.98	1.00	0.96	0.99	0.98
9	2	Pos/neg	Yes	No	Fresh	ROC	289	0	675.3	701.0			0.34	0.96	0.97	0.87	0.96	0.91	0.99
10	2	Pos/neg	Yes	No	Dried	ROC	289	0	647.8	673.5			0.33	0.96	0.96	0.84	0.99	0.94	0.99
11	2	Pos/neg	Yes	No	Fresh	MM	289	0	676.3	702.0			0.36	0.95	0.99	0.99	0.89	0.88	0.99
12	2	Pos/neg	Yes	No	Dried	MM	289	0	633.0	658.7			0.35	0.95	0.98	0.99	0.95	0.90	0.99
13	2	Pos/ind/neg	Yes	Yes	Fresh	ROC	297	48	1102.9	1187.9	0.15	< 0.01	0.39 (0.33–0.44)	0.89 (0.82–0.95)	0.97 (0.94–0.99)	0.70 (0.60–0.79)	0.97 (0.95–1.00)	0.77 (0.69–0.86)	0.96 (0.92–0.99)
14	2	Pos/ind/neg	No	Yes	Fresh	ROC	579	13	1632.2	1688.9	< 0.01	< 0.01	0.42 (0.38–0.47)	0.95 (0.91–0.98)	0.97 (0.95–0.99)	0.80 (0.74–0.86)	0.96 (0.94–0.99)	0.97 (0.94–0.99)	0.96 (0.94–0.99)
15	2	Pos/ind/neg	Yes	No	Fresh	ROC	297	13	956.0	1004	< 0.01	< 0.01	0.39 (0.32–0.45)	0.89 (0.81–0.96)	0.96 (0.93–1.00)	0.70 (0.59–0.80)	0.97 (0.94–1.00)	0.80 (0.71–0.89)	1.00 (0.98–1.00)
16	3	Pos/ind/neg	Yes	Yes	Fresh	ROC	297	36	1096.4	1225.7	0.84	0.04	0.27 (0.11–0.43)	0.99 (0.91–1.00)	0.97 (0.94–1.00)	0.92 (0.63–1.00)	0.98 (0.95–1.00)	0.89 (0.79–0.99)	0.96 (0.93–0.99)
**17**	**3**	**Pos/ind/neg**	**No**	**Yes**	**Fresh**	**ROC**	**579**	**6**	**1613.4**	**1700.6**	**0.85**	**0.95**	**0.38 (0.30–0.46)**	**0.98 (0.96–1.00)**	**0.98 (0.96–1.00)**	**0.90 (0.73–1.00)**	**0.97 (0.95–0.99)**	**0.98 (0.96–1.00)**	**0.96 (0.94–0.99)**
18	3	Pos/ind/neg	Yes	No	Fresh	ROC	297	6	939.5	1013.4	0.09	0.04	0.35 (0.27–0.42)	0.95 (0.89–1.00)	0.99 (0.96–1.00)	0.78 (0.65–0.91)	0.98 (0.94–1.00)	0.84 (0.75–0.94)	1.00 (0.98–1.00)
19	2	Pos/ind/neg	Yes	Yes	Dried	ROC	297	48	1129.1	1214.1	0.03	< 0.01	0.39 (0.33–0.44)	0.89 (0.82–0.96)	0.96 (0.94–0.99)	0.61 (0.51–0.72)	0.97 (0.94–1.00)	0.78 (0.70–0.86)	0.95 (0.92–0.99)
20	2	Pos/ind/neg	No	Yes	Dried	ROC	579	13	1675.4	1732.1	< 0.01	< 0.01	0.42 (0.38–0.47)	0.95 (0.92–0.99)	0.97 (0.95–0.99)	0.75 (0.69–0.82)	0.97 (0.94–0.99)	0.97 (0.94–1.00)	0.96 (0.93–0.98)
21	2	Pos/ind/neg	Yes	No	Dried	ROC	297	13	976.4	1024.4	< 0.01	< 0.01	0.37 (0.31–0.43)	0.90 (0.82–0.97)	0.94 (0.90–0.99)	0.63 (0.52–0.75)	0.96 (0.93–1.00)	0.84 (0.75–0.92)	1.00 (0.98–1.00)
**22**	**3**	**Pos/ind/neg**	**Yes**	**Yes**	**Dried**	**ROC**	**297**	**36**	**1109.8**	**1239.1**	**0.92**	**0.93**	**0.27 (0.18–0.37)**	**0.99 (0.96–1.00)**	**0.97 (0.94–1.00)**	**0.86 (0.59–1.00)**	**0.98 (0.95–1.00)**	**0.89 (0.82–0.96)**	**0.96 (0.93–0.99)**
**23**	**3**	**Pos/ind/neg**	**No**	**Yes**	**Dried**	**ROC**	**579**	**6**	**1650.0**	**1737.2**	**0.45**	**0.62**	**0.37 (0.27–0.47)**	**0.99 (0.98–1.00)**	**0.98 (0.96–1.00)**	**0.86 (0.66–1.00)**	**0.97 (0.95–1.00)**	**0.98 (0.96–1.00)**	**0.97 (0.93–1.00)**
24	3	Pos/ind/neg	Yes	No	Dried	ROC	297	6	951.8	1025.7	0.09	0.17	0.30 (0.21–0.38)	0.98 (0.95–1.00)	0.99 (0.93–1.00)	0.79 (0.60–0.98)	0.99 (0.95–1.00)	0.90 (0.82–0.99)	1.00 (1.00–1.00)

These three models estimated similar sensitivities and specificities for all three tests. The best-fitting model to describe the data from pre-coated plates appeared to be Model 23, which included the results from LFA testing with serum but not blood. This model estimated sensitivity for MBA, ELISA, and LFA to be 0.99 (95% CI: 0.98–1.00), 0.86 (0.66–1.00), and 0.98 (0.96–1.00), respectively. Specificity for MBA, ELISA, and LFA in the same model was 0.98 (0.96–1.00), 0.97 (0.95–1.00), and 0.97 (0.93–1.00), respectively. Compared to the sensitivity and specificity estimates for the MBA and ELISA derived from comparisons to NAAT^13^, the sensitivities and specificities derived using best-fitting latent class model were similar (Table 1). The point estimates for sensitivity and specificity for ELISA were lower in the latent class model than those based on comparisons to NAAT, but due to the latent class model’s wide confidence intervals, these were not significantly different. Generally, estimates from the latent class models contained more uncertainty than estimates from the NAAT analyses. Specificities obtained using non-endemic controls compared to latent class analysis were 0.97 (0.95–1.00) versus 0.98 (0.96–1.00) for MBA, 0.98 (0.96–1.00) versus 0.97 (0.95–0.99) for ELISA, and 0.99 (0.98–1.00) versus 0.96 (0.94–0.99) for LFA (Table 1).

The proportion estimated as positive was quite similar for the models that included either freshly-coated plates or dried plates with only LFA serum tests (0.38 [0.30–0.46] and 0.37 [0.27–0.47], respectively) but was somewhat, though not significantly lower, when using both LFA tests and dried plates (0.27 [0.18–0.37], Table 1).

### Evaluating model fit

Many models did not have greater than zero degrees of freedom or fit the data well based on the G^2^ and Pearson χ^2^ tests (Table 1). The degrees of freedom in a latent class model is determined by the number of test result combinations (also called patterns) minus the number of model parameters minus one. When the degrees of freedom are less than or equal to zero, we are fitting a model that has at least as many parameters to estimate as there are patterns. Under such circumstances, maximum likelihood estimates of sensitivity and specificity are provided by the software, but these results should be regarded with caution. Also, none of the sensitivity and specificity values have confidence intervals because positive degrees of freedom are required in that calculation.

For some selected models, we explored the model fit further by looking at each pattern’s contribution to the G^2^ and Pearson χ^2^ test statistics. The results for two models are presented in Table 2: both included the indeterminate category, and used results from ELISA testing with pre-coated plates, and results of LFA tests for both blood and serum, but one model included two latent classes (Model 19 from Table 1) and the other included three latent classes (Model 22 from Table 1). Based on the statistical tests, the fit for the model with two latent classes was poor (G^2^_(48)_ = 68.03, p = 0.03; χ^2^_(48)_ = 144.72, p < 0.01) while model with three latent classes had the best fit (G^2^_(36)_ = 24.73, p = 0.92; χ^2^_(36)_ = 24.21, p = 0.93). The difference in fit can be largely explained by the two-latent-class model having difficulty with patterns containing indeterminate values. For instance, the largest contribution to the G^2^ statistic in the two-latent-class model is the pattern where results of MBA and ELISA are indeterminate, the LFA blood sample was negative, and the LFA serum sample was positive. The model expects only 0.26 people to have this pattern, but three were observed with that pattern. When a third latent class is added in the second model, the model predicts that 1.16 people will have that pattern, which greatly reduces the contribution to the fit statistics. Also, the predicted classification changes from positive (probability of 0.99) in the two class model to indeterminate (probability of 1.00) in the three class model.

**Table 2:** Contribution of each pattern to the G^2^ and Pearson chi-squared test statistics. “+” = positive, “−” = negative, “?” = indeterminate. LFA = lateral flow assay; ELISA = enzyme-linked immunosorbent assay; MBA = multiplex bead array. Model with two latent classes utilizes Model 19 from Table 1 and Model with three latent classes utilizes Model 22 from Table 1.

					Model with two latent classes					Model with three latent classes					
MBA	ELISA	LFA (blood)	LFA (serum)	N	Estimated probability positive	Estimated probability negative	Expected N	Contribution to χ^2^	Contribution to G^2^	Estimated probability positive	Estimated probability negative	Estimated probability indeterminate	Expected N	Contribution to χ^2^	Contribution to G^2^
—	—	—	—	164	0.00	1.00	162.63	0.01	2.75	0.00	1.00	0.00	164.10	0.00	−0.21
—	—	—	?	3	0.00	1.00	3.01	0.00	−0.01	0.00	0.96	0.04	2.90	0.00	0.20
—	—	—	+	4	0.01	0.99	3.86	0.01	0.29	0.00	0.88	0.12	3.86	0.01	0.29
—	—	+	+	1	0.37	0.63	1.06	0.00	−0.11	0.00	0.00	1.00	1.04	0.00	−0.07
—	?	—	—	4	0.00	1.00	5.28	0.31	−2.21	0.00	0.97	0.03	3.97	0.00	0.06
−	?	?	+	1	1.00	0.00	0.06	15.01	5.66	0.01	0.00	0.99	0.32	1.43	2.27
−	?	+	−	1	0.39	0.61	0.03	31.36	7.01	0.00	0.00	1.00	0.14	5.44	3.98
−	?	+	+	1	0.97	0.03	0.78	0.06	0.50	0.10	0.00	0.90	1.35	0.09	−0.60
−	+	+	+	1	1.00	0.00	1.81	0.37	−1.19	1.00	0.00	0.00	0.86	0.02	0.31
?	−	−	−	3	0.00	1.00	3.83	0.18	−1.46	0.00	0.93	0.07	3.05	0.00	−0.10
?	−	+	+	3	0.98	0.02	0.93	4.61	7.03	0.00	0.00	1.00	2.19	0.30	1.88
?	?	−	−	1	0.00	1.00	0.12	6.19	4.17	0.00	0.22	0.78	0.31	1.55	2.36
?	?	−	?	1	0.94	0.06	0.04	24.35	6.54	0.00	0.00	1.00	0.30	1.60	2.39
?	?	−	+	3	0.99	0.01	0.26	29.01	14.70	0.00	0.00	1.00	1.16	2.91	5.69
?	?	+	+	2	1.00	0.00	1.77	0.03	0.49	0.00	0.00	1.00	2.58	0.13	−1.02
+	−	−	−	2	0.00	1.00	2.08	0.00	−0.15	0.00	0.77	0.23	1.99	0.00	0.02
+	−	−	?	1	0.85	0.15	0.25	2.27	2.78	0.00	0.04	0.96	0.60	0.26	1.01
+	−	−	+	3	0.97	0.03	1.57	1.30	3.89	0.01	0.01	0.98	2.26	0.24	1.69
+	−	?	?	1	1.00	0.00	0.12	6.59	4.27	0.00	0.00	1.00	0.28	1.88	2.56
+	−	?	+	2	1.00	0.00	0.82	1.68	3.55	0.01	0.00	0.99	1.29	0.39	1.74
+	−	+	+	5	1.00	0.00	10.52	2.89	−7.43	0.04	0.00	0.96	5.14	0.00	−0.28
+	?	−	+	2	1.00	0.00	2.94	0.30	−1.55	0.22	0.00	0.78	3.33	0.53	−2.04
+	?	?	+	2	1.00	0.00	1.59	0.10	0.91	0.17	0.00	0.83	1.83	0.02	0.36
+	?	+	−	1	1.00	0.00	0.32	1.48	2.30	0.00	0.00	1.00	0.65	0.18	0.85
+	?	+	+	16	1.00	0.00	20.31	0.92	−7.64	0.62	0.00	0.38	15.32	0.03	1.40
+	+	−	+	5	1.00	0.00	7.04	0.59	−3.42	1.00	0.00	0.00	4.64	0.03	0.74
+	+	?	+	2	1.00	0.00	3.81	0.86	−2.58	1.00	0.00	0.00	2.01	0.00	−0.03
+	+	+	?	1	1.00	0.00	0.54	0.38	1.22	1.00	0.00	0.00	0.85	0.03	0.32
+	+	+	+	61	1.00	0.00	48.59	3.17	27.75	1.00	0.00	0.00	61.53	0.00	−1.05

In large part, the contributions to both test statistics are similar, except for indeterminate test results. Both models performed well at predicting the number of samples with each pattern, except for patterns with indeterminate values, and especially with indeterminate ELISA values. The largest contributions to the fit statistics are those patterns with indeterminate ELISA test results; these are the patterns that drive the two-class model to have a poor fit.

### Number of latent classes and assay cutoffs

The best fitting models were those with three latent classes and indeterminate values included (Table 1). Models without either of those (two latent classes or without an indeterminate category) had insufficient degrees of freedom or the G^2^ or Pearson chi-squared test was rejected.

Across models, regardless of whether or not they fit the data well, when the same methodology was used (ROC) for determining cutoffs for ELISA and MBA, the sensitivity and specificity of the different platforms were generally high. In general, there was higher sensitivity for MBA and ELISA and higher specificity for LFA, regardless of the number of test criteria or classes (Table 1). In otherwise similar models, the sensitivity of the LFA tended to be lower using whole blood than using serum (Table 1). For instance, the LFA sensitivity when using both blood and serum, three latent classes, and pre-coated ELISA plates was 0.89 (0.82–0.96, Model 22). A similar sensitivity of 0.90 (0.82–0.99) was found with blood only (Model 24), but this increases to 0.98 (0.96–1.00) when only serum LFA test results are included (Model 23).

Specificity for MBA and ELISA was consistent across most models, generally above 0.95. Sensitivity for MBA and ELISA varied. MBA sensitivity was generally higher (but not significantly so) when only the serum LFA test was used and similar when the blood LFA test and when both LFA tests were used. ELISA sensitivity was generally lowest when only the blood LFA test was included and similar in the two other models.

Using the mixture model to determine cutoffs for the ELISA affected its sensitivity and specificity but this had little effect on the other tests in the analysis (Table 1). ELISA sensitivity dropped from 0.99 (0.96–1.00, Model 4) with mixture model cutoffs to 0.81 (0.69–0.92, Model 2) with ROC cutoffs when using both blood and serum LFA and two latent and diagnostic classes. For the same comparison, the ELISA specificity increased slightly, but non-significantly, from 0.96 (0.92–1.00, Model 4) to 0.99 (0.97–1.00, Model 2).

### LFA blood or serum

Of the three best fitting models, two included only the LFA test using serum, while the third included both blood and serum LFA tests. No models with blood only appeared to fit the data well.

### Pre-coated or freshly-coated plates

For models including results from ELISA testing using pre-coated plates, fit for models using serum-only LFA models and blood and serum LFA was adequate. For models including results from ELISA testing using freshly-coated plates, the only model that had an adequate fit by both tests was the model including only the serum LFA test. In the best fitting latent class model using ELISA testing with freshly-coated plates, sensitivities were 0.98 (0.96–1.00) for MBA, 0.90 (0.73–1.00) for ELISA, and 0.98 (0.96–1.00) for LFA.

### Models including age and sex as covariates

For models including age and sex as covariates, fit for the model including blood and serum for LFA was adequate (model S1, Supplemental Table 1). There was no change in sensitivity or specificity when including age and sex as covariates in the analysis. The proportion positive increased, likely due to the loss of the Bolivian sample set (for which data were anonymized prior to these analyses). The model including the parameters of model S1 was rerun without age and sex as covariates using the same sample set (model S2, Supplemental Table 1). Comparing models S1 and S2, the only substantial difference seen is in the ELISA sensitivity estimate, although the confidence intervals for both estimates are wide and overlap.

## Discussion

The sensitivities and specificities estimated from the best-fitting latent class model were generally similar to estimates of those parameters based on NAAT testing as a gold standard. Sensitivity estimates differed by at most seven hundredths and confidence limits were overlapping in each case. Specificities were more similar with differences of no more than three hundredths. One major difference between those models were the confidence intervals for ELISA. In the NAAT comparisons, the confidence interval stretched from 0.88 to 0.98. For the latent class model, that interval stretched from 0.73 to 1.00. Confidence intervals were generally larger in the latent class models as compared to the comparisons to NAAT, suggesting that the lack of a gold standard may increase the uncertainty in the results.

Based on the latent class analysis, LFA has lower sensitivity when using whole blood compared to using serum. In this study the “whole blood” used was created by mixing serum with blood cells depleted of serum from an uninfected individual^13^. While this has the advantage of directly comparing these two tissue types from the same specimen, it is possible that the performance of this manufactured whole blood is affected by the processing (e.g., centrifugation) that would not occur in field settings. Field studies are currently underway to determine if this difference in sensitivity holds when testing whole blood directly. If so, efforts to improve the sensitivity of the LFA using whole blood will be undertaken.

Including an indeterminate category for the results of diagnostic tests improved the model fit. Any model with only positive and negative diagnostic categories failed to achieve a satisfactory model fit. This underscored the utility of having more patterns in the dataset. The impact of adding the indeterminate categories concurs with simulations which show having more, higher-quality indicators produces a better model^15^. The indeterminate latent class acknowledges that there is more uncertainty around this measurement than for those measurements falling outside of the indeterminate range (for quantitative assays) or for which there was agreement between readers (for dichotomoized tests). Biologically it suggests low-level antibody responses, likely due to mild infection, declining responses, or low-level cross-reactivity with antibodies arising against non-CT antigens. In most cases, having four or fewer indicators resulted in low-quality latent class models, defined by simulations where at least 50% of the replications did not converge, possessed inaccurate group assignment, labeled groups incorrectly, or estimated zero-variance for an observed variable. In studies, it is often not feasible to have this many diagnostic tests either because no more than four tests exist, it is too costly to use multiple tests in field settings, or the tests are not conditionally independent. Regardless, investigators planning to use latent class models to assess the sensitivity and specificity of tests where no gold standard exists should use as many tests as are available, even if they may be correlated. Adding tests that are not conditionally independent is slightly more complicated because those tests need to be combined into categories, but our case study here shows the increase in the number of patterns (combinations of diagnostic test results) results in a more robust model.

Models that included age and sex as covariates tended to have poor fits, although that may be because of the lower N due to the exclusion of the anonymized Bolivia sample set that lacked age and sex information. The model with the best fit utilized 3 latent classes, 3 diagnostic categories, and the ELISA freshly coated plates with a ROC cutoff. The inclusion of age and sex as covariates when the same sample sets were included in the models (model S1) showed little variation to the models excluding age and sex (model S2). Only the ELISA sensitivity was affected by age and sex as covariates, although the confidence intervals for ELISA sensitivity are quite wide, making interpretation difficult. Age and sex seem to have little overall effect on the performance of assays measuring responses against Pgp3 in this analysis.

An additional latent class also made a large difference in the model fits. Commonly, diagnostic test studies use two latent classes since the goal is classify individuals as positive or negative. Including an additional category appeared to improve the model fit because results that are difficult to classify, especially those that are indeterminate, can be included. One limitation of including an indeterminate class is that computing sensitivity and specificity becomes problematic, since a 2 × 2 table is assumed. We chose the most conservative route which was to consider that group an incorrect classification when calculating both sensitivity and specificity, but better options may be available.

The results in Table 2 also highlight the benefits of a thorough assessment of model fit. Using the fit statistics eliminated many potential models and provided some general clues as to why certain models were having trouble. After exploring the contributions to the G^2^ and Pearson chi-squared test statistics for two models, the addition of another latent class proved very important.

The latent class models presented here produced similar sensitivity and specificity estimates compared to prior analyses that treated NAAT results as a gold standard or assumed negative results as correct for non-endemic control samples. Sensitivity and specificity estimates from latent class models possessed wider confidence intervals than previous comparisons^13^, suggesting analyses which properly account for the lack of a gold standard may result in greater uncertainty. These and other analyses using diagnostic tests without a gold standard would benefit from having more tests included. These analyses underscored the need to evaluate latent class model fit.

## Methods

### Laboratory Methods

Full treatment of the laboratory methods can be found elsewhere^13^. We analyzed results of testing of serum samples from 579 individuals from three populations. One set was collected as part of a trachoma serosurveillance study of all ages in a community in Nepal (N = 424)^16^. Two sets were collected as non-endemic controls: one of 4–9-year-olds as part of a Chagas disease study from three communities in central Bolivia (N = 81) and another of 1–9-year-olds from Brooklyn, NY on a pediatric panel (N = 74).

Full descriptions of the selection and isolation of the CT antigen Pgp3^6^, details of the MBA and ELISA processes^13^, and the LFA test^12,13^ are available elsewhere.

#### Ethics

Samples were collected under approved protocols^13^. For Nepalese samples, IRB approval was obtained from Children’s Hospital and Research Center Oakland (IRB number 2013-043) and by Nepal Netra Jhoti Sangh (Nepali Prevention of Blindness Program) and data were not anonymized for these researchers. CDC researchers were non-engaged (i.e., did not have access to patient identifying information) in the study. All study participants gave written informed consent, or written parental consent was obtained for participants under the age of 18. For Bolivian samples, approval was obtained from the Asociacion Beneficia PRISMA and from CDC. Samples were stripped of identifying information after these studies were terminated, and additional testing conducted in accordance with consents obtained for each study. Samples from Brooklyn were obtained anonymously; approval was obtained from the local Institutional Review Boards (protocol #412878). The work described here was approved as non-human subjects research by the Office of the Associate Director for Science, Center for Global Health at CDC. All experiments were performed in accordance with the guidelines of approving institutions. Informed consent was obtained from individuals over 17 years of age and parental consent given for individuals aged 1–17.

#### Latent Class Analysis

Maximum likelihood via the expectation-maximization (EM) algorithm^17^ was used to find the optimal probabilities of latent class assignment which best fit the observed data. As required for latent class analysis, the total number of classes was chosen a priori. Further details on the fitting process can be found elsewhere^2,18,19^.

#### Modeling choices

These analyses of trachoma antibody test data presented multiple options for a latent class model. Below we outline considerations related to modeling the trachoma data. Some of these options are needed for all latent class analyses, while others are specific to the current analyses:

1. Sample size
  A first consideration for all latent class analyses is an appropriate sample size. Sample size estimates vary largely, which may be attributable to dependencies on the quality and number of tests included^15^. For these analyses, we set a goal for 500 samples. We exceeded this goal with 579 serum samples, but were only able to obtain 297 blood samples.
  We used our full sample size of 579 when indeterminate results from the ELISA and LFA serum assays were included. With indeterminate results omitted, the sample size dropped to 569. Models that include LFA blood tests have a sample size of 297. This sample size drops to 289 for analyses when indeterminate LFA results are excluded and to 283 for analyses that include LFA tests run on both blood and serum.
2. Choice of model
  Many types of approaches use latent variables including latent class analysis, latent profile analysis, factor analysis, and item response theory. All are a subset of structural equation models. Since the LFA test is dichotomous and the latent variable (disease status) is categorical, latent class analysis was most appropriate for this analysis. We used a method for polytomous outcome variables^20^ to estimate the performance of each test in the absence of a gold standard.
3. Number of latent classes
  For all latent class models, one must decide the number of latent classes and whether to include covariates. A model fitting two latent classes would indicate classes of negative and positive, while three latent classes would indicate positive, indeterminate, and negative. We did not fit higher than three latent classes because we were unsure how to interpret additional latent classes. The only covariate considered for models was age.
4. Evaluating model fit
  Model fits were evaluated based on the Akaike Information Criterion (AIC)^21^, the Bayesian Information Criterion (BIC)^22^, Pearson χ^2^ goodness of fit and likelihood ratio χ^2^ (G^2^) statistics^23^. The former two are unitless measures of model fit. The latter two tests have a null hypothesis that the model adequately fits the data, hence, a small p-value indicates a poorly fitting model. There is debate on the accuracy of these tests^24^, however, making fit evaluation with these metrics somewhat tricky. For these analyses, we focused on models with large p-values that would provide evidence of an adequate fit even if the tests are underpowered. We also considered models with lower AIC and BIC, but comparisons can only be made between models with the same dependent variable, limiting the usefulness. Analyses were performed in R version 3.2.2^25^ using the poLCA package^19^. From the models, we output the estimated sensitivity, specificity, and 95% confidence intervals for each assay.
5. Assay cutoffs
  For MBA assays, Pgp3 antigen was chemically conjugated to microspheres (i.e. “coupled”) as the capture reagent in the assay. Two different sets of Pgp3 couplings to beads were used for the MBA testing. Coupling 1 was used with all serum samples from Nepal (N = 424) and coupling 2 was used with all non-endemic controls (N = 155). The cutoff for coupling 1 was determined by receiver operator characteristic (ROC) curves using non-endemic pediatric samples from Milwaukee (N = 116) as a non-exposed population^26^ and NAAT positives from Tanzania (N = 41). This cutoff was estimated to be a median fluorescence intensity (MFI) with background subtracted (MFI-BG) of 801 by taking the midpoint between the highest MFI-BG value of the “negative” samples and the lowest MFI-BG value of the “positive” samples in the ROC panel. The indeterminate range —calculated by adding and subtracting 20% of the cutoff — was 641 to 961. The cutoff for coupling 2 and the ELISA were generated using ROC curves from a panel of 66 previously classified MBA positive and negative samples. The cutoff for coupling 2 was 804 with an indeterminate range of 643–965. The ELISA cutoffs were estimated to be an optical density (OD) of 0.983 and 1.104 for freshly coated and pre-coated plates (see below), respectively. The indeterminate ranges were 0.786–1.180 for freshly-coated plates and 0.883–1.325 for pre-coated plates. When using the indeterminate range, we trichotomized the assay data where observations above the upper limit were treated as positive, those between the limits were indeterminate, and those below the lower limit were negative.
  For ELISA data, we also used a mixture model cutoff based on the formula $\mu +3\ast \sigma$ where $\mu$ and $\sigma$ are the estimated mean and standard deviation of the negative distribution of the study data^27^. Participants with a value above a cutoff were determined positive for that assay measurement and method and those below were negative. LFA data were classified as positive when both readers determined the sample to be positive and negative when both read the sample as negative. When there was disagreement between readers, results were considered indeterminate.
6. Specimen type run on LFA (blood vs serum)
  We ran models using the LFA blood and serum tests individually and together. Including each as a single indicator is unlikely to meet the conditional independence assumption, so we created a categorical variable with each possible combination of results from the blood and serum tests. For instance, people with positive results on both tests are grouped into one category, those with a positive LFA blood test and a negative LFA serum test into another category, participants with a negative LFA blood test and a positive LFA serum test groups into another category, and so on.
7. Pre-coated or freshly-coated ELISA plates
  We ran a set of analyses with the ELISA data from plates that had been pre-coated with antigen and another with plates freshly coated with antigen (the standard method for Pgp3 assays to date) to compare the results.
8. Age and sex as covariates

Finally, we ran a set of analyses in which age and sex were included as covariates. As no age or sex data were available for the samples from Bolivia (which had previously been anonymized), an additional model was run using the same parameters but without age and sex as covariates to allow for comparisons to be made on the same data.

### Data availability

Unidentified raw data from analyses are available upon request.

## Electronic supplementary material

Supplementary Table 1
