# Supplementary material for: Latent class modeling to compare testing platforms for detection of antibodies against the Chlamydia trachomatis antigen Pgp3
Source: Sci Rep. 2018 Mar 9;8:4232. doi: 10.1038/s41598-018-22708-9 (PMC5844876; doi:10.1038/s41598-018-22708-9)
Supplement: Supplementary file 1 — Supplementary Table 1 [file 41598_2018_22708_MOESM1_ESM.docx]

**Latent class modeling to compare testing platforms for detection of antibodies against the *Chlamydia trachomatis* antigen Pgp3**

Ryan E. Wiegand^1^, Gretchen Cooley^1^, Brook Goodhew^1^, Natalie Banniettis^2^, Stephan Kohlhoff^2^, Sarah Gwyn^1^, Diana L. Martin*^1^

Affiliations:

1 Division of Parasitic Diseases and Malaria, Centers for Disease Control and Prevention, Atlanta GA, USA

2 State University of New York Downstate Medical Center, Brooklyn, NY, USA

*Corresponding Author:

Diana L Martin

MS D-65

1600 Clifton Rd NE

Atlanta Ga USA 30329

404-718-4147 (phone)

404-718-4193 (fax)

[hzx3@cdc.gov](mailto:hzx3@cdc.gov)

| model # | Latent Classes | Diagnostic categories | LFA blood test | LFA serum test | ELISA coating | Cutoff | N | DF | AIC | BIC | G^2^ test p-value | χ^2^ test p-value | Proportion positive | MBA Sensitivity | MBA Specificity | ELISA Sensitivity | ELISA Specificity | LFA Sensitivity | LFA Specificity |
| --- | --- | --- | --- | --- | --- | --- | --- | --- | --- | --- | --- | --- | --- | --- | --- | --- | --- | --- | --- |
| 16 | 3 | Pos/ind/neg | Yes | Yes | Fresh | ROC | 297 | 36 | 1096.4 | 1225.7 | 0.84 | 0.04 | 0.27 (0.11-0.43) | 0.99 (0.91-1.00) | 0.97 (0.94-1.00) | 0.92 (0.63-1.00) | 0.98 (0.95-1.00) | 0.89 (0.79-0.99) | 0.96 (0.93-0.99) |
| **S1** | **3** | **Pos/ind/neg** | **Yes** | **Yes** | Fresh | ROC | **212** | **32** | **937.4** | **1068.3** | **0.75** | **0.08** | **0.37 (0.23-0.52)** | **0.95 (0.88-1.00)** | **0.95 (0.90-1.00)** | **0.96 (0.62-1.00)** | **0.98 (0.95-1.00)** | **0.87 (0.78-0.96)** | **0.92 (0.87-0.98)** |
| S2 | 3 | Pos/ind/neg | Yes | Yes | Fresh | ROC | 212 | 36 | 944.2 | 1061.7 | 0.9 | 0.37 | 0.42 (0.33-0.51) | 0.94 (0.88-1.00) | 0.95 (0.90-1.00) | 0.85 (0.70-1.00) | 0.98 (0.95-1.00) | 0.87 (0.78-0.95) | 0.92 (0.86-0.98) |

**Supplemental Table 1**  Sensitivity and specificity of tests measuring antibodies to Pgp3 based on input parameters with an without age and sex covariates included. Pos = positive; ind = indeterminate; neg = negative. LFA = lateral flow assay; ELISA = enzyme-linked immunosorbent assay; MBA = multiplex bead array. ROC = receiver operator characteristics; MM = mixture model. DF = degrees of freedom. AIC = Akaike Information Criterion. BIC = Bayesian Information Criterion. Bolded rows are referenced in the text
